# A predictive model for advanced oropharyngeal cancer patients treated with chemoradiation

**DOI:** 10.1186/s12885-022-09732-9

**Published:** 2022-06-05

**Authors:** Wu-Chia Lo, Chih-Ming Chang, Chia-Yun Wu, Chen-Hsi Hsieh, Pei-Wei Shueng, Po-Wen Cheng, Li-Jen Liao

**Affiliations:** 1 Department of Otolaryngology, Far Eastern Memorial Hospital, 21, Section 2, Nan-Ya South Road, Banqiao, Taipei, 22061 Taiwan; 2 Graduate Institute of Medicine, Yuan Ze University, Taoyuan, Taiwan; 3 Head and Neck Cancer Surveillance and Research Study Group, Far Eastern Memorial Hospital, New Taipei City, Taiwan; 4 Department of Biomedical Engineering, National Yang-Ming Chiao Tung University, Taipei, Taiwan; 5 Department of Oncology and Hematology, Far Eastern Memorial Hospital, Taipei, Taiwan; 6 Department of Radiology, Division of Radiation Oncology, Far Eastern Memorial Hospital, Taipei, Taiwan; 7 Department of Medicine, School of Medicine, National Yang-Ming Chiao Tung University, Taipei, Taiwan; 8 Department of Electrical Engineering, Yuan Ze University, Taoyuan, Taiwan

**Keywords:** Oropharyngeal cancer, Death, Hemoglobulin, Systemic immune inflammation, Nomogram, Chemoradiation, Survival

## Abstract

**Background:**

To analyze clinical characteristics in the prediction of death within 1 year in advanced oropharyngeal cancer patients treated with chemoradiation.

**Methods:**

One hundred forty-seven advanced oropharyngeal cancer patients who underwent curative-intent chemoradiation treatment were retrospectively enrolled. The pre-treatment clinical parameters including inflammatory markers were reviewed.

**Results:**

The 1-year death rate for all patients was 29% [95% confidence interval (CI): 23–37%]. In multivariate logistic regression analysis, hemoglobulin (Hb) < 13.5 g/dl was an independent indicator of death within 1-year [Odds ratio (OR) 5.85, 95% CI 2.17–15.75, *p* < 0.001]. Systemic immune inflammation (SII) ≥ 1820 was also a significant factor for prediction of death within 1 year (OR 4.78, 95% CI 1.44–15.85, *p* = 0.011). We further used gander, age, Hb and SII to develop a nomogram to predict death within 1 year. The c-index of the model was 0.75 (95%CI 0.66–0.83). For patients with low nomogram score (< 14) versus high nomogram score (≥ 14), the 1-year and 2-year OS rates were 91 and 71% versus 53 and 29%, respectively. (*p* < 0.001). A difference in the disease persistence or recurrence rate between patients with high and low nomogram score was significant (73 and 28%, respectively; *p* < 0.001).

**Conclusions:**

The pre-treatment Hb < 13.5 g/dl and SII ≥ 1820 are associated with higher risks of death within 1-year in patients with advanced oropharyngeal cancers. Nomogram can aid in patient counseling and treatment modality adjustment. The development of a more effective treatment protocol for patients with high nomogram score will be essential.

**Supplementary Information:**

The online version contains supplementary material available at 10.1186/s12885-022-09732-9.

## Background

The worldwide incidence rates (cumulative risk) of oropharyngeal cancer for men and women are 0.21 and 0.05%, respectively [1]. Despite improvements in the management outcomes of human papillomavirus (HPV) associated patients, a noteworthy percentage of newly-diagnosed oropharyngeal cancer seem to be driven by traditional carcinogen, such as tobacco and alcohol [2]. In advanced oropharyngeal cancer, concurrent chemoradiation with/without neoadjuvant chemotherapy are used as a standard treatment for whom an organ-preservation strategy is desirable [2]. However, some patients still display a poorer overall survival and experience early death (within 1 year) after standard treatment. Therefore, the identification of biomarkers to determine prognosis can help us to adjust treatment for specific patient subgroups and potentially improve disease control.

In recent years, tumor oxygenation and antitumoral immunity are two main issues related to the tumor microenvironment that are progressively explored to elucidate the higher sensitivity to chemoradiation and the better outcomes, as well as in the view of evolving new diagnosis and prognosis tools [3, 4]. Previous literature have showed that low hemoglobulin is associated with worse local control and survival [5–8]. Low hemoglobulin is supposed to lead to decreased cell oxygenation and contribute to chemoradiation resistance via oxygen deprivation [9, 10]. Inflammatory responses in tumor microenvironment can be reflected by some common markers in peripheral blood, such as lymphocytes, neutrophils, monocytes, and platelets [11–17]. Increased level of lymphocyte has been appeared to be involved in the host immune response against the growth and metastasis of cancer cells [18], while tumor-infiltrating neutrophils are key mediators in promoting tumor growth and metastasis [19, 20]. The lymphocyte-to-monocyte ratio (LMR), the neutrophil-to-lymphocyte ratio (NLR), the platelet-to-lymphocyte ratio (PLR), systemic inflammation response index (SIRI), and systemic immune inflammation (SII) are emerging markers of host inflammation that predict the prognosis in head and neck cancer patients [21, 22]. A combined analysis of the peripheral blood counts of neutrophils, monocytes and lymphocytes have been proposed to be SIRI; while SII is a combined analysis of the peripheral blood counts of platelet, neutrophils and lymphocytes [21, 22].

This study was to assess and elucidate the pre-treatment prognostic markers that predict death within 1-year in advanced oropharyngeal cancer patients treated with curative-intent chemoradiation therapy with/without neoadjuvant chemotherapy. We further developed a nomogram to predict death within 1-year of advanced oropharyngeal cancer patients in our large single institutional cohort.

## Methods

The study was approved by the Institutional Review Board (Far Eastern Memorial Hospital 109,169-E) in a tertiary hospital. The need of informed consent was waived by Research Ethics Review Committee of Far Eastern Memorial Hospital (No.: 109169-E) due to retrospective and anonymous study design. We confirmed that all methods were performed in accordance with the Declaration of Helsinki. Patients diagnosed with advanced (stage III, IVa, and IVb) oropharyngeal cancer from Feb. 2008 to Nov. 2019 were retrospectively reviewed. Patients were staged according to the 7th edition American Joint Committee on Cancer Staging Manual (AJCC). All patients met the following criteria: (a) histologically confirmed primary squamous cell carcinoma; (b) no evidence of distant metastasis; and (c) received definite chemoradiation with/without neoadjuvant chemotherapy. The standard concomitant therapy consisted of cisplatin 30–35 mg/m^2^ in one-week interval for all patients with radiation therapy (RT) 70 Gy divided in 35 fractions treated for 7 weeks. In few cases of neoadjuvant (induction) chemotherapy, docetaxel, cisplatin, and fluorourocil were prescribed. All recruited patients had completed the treatment course and follow-up till death or Dec. 2020.

Medical records were obtained from the patients’ charts and the institutional cancer registration system including: pre-treatment laboratory data, demographic information, smoking status, human papillomavirus (HPV) status, the location and extent of the local and/or regional disease, the disease persistence after treatment, the recurrence (local, regional and distant) time and localization, death date and date of the last follow-up. Persistent disease was defined as tumor was detected by the imaging methods 3 months after treatment. Tumor recurrence was defined as structural disease diagnosed more than 3 months after treatment in patients without persistent disease. All blood tests were performed within 2 weeks prior to standard treatment. Strong and diffuse (> 75% of tumor cells) p16 immunohistochemistry staining was classified as HPV positive.

### Statistical analysis

Data are expressed as the mean ± standard deviation (SD), median and interquartile range (IQR), percent (%), odds ratio (OR) and 95% confidence interval (CI) where appropriate. Two-sided student’s T test was performed for continuous variables. Fisher’s exact or Chi-squared test was used as appropriate for categorical variables. The overall survival (OS) duration was recorded from the completion date of the comprehensive treatment to the date of death or final visit. Early death was defined as the time interval between the final treatment day and the date of death within 1 year. Early death has different definitions ranging from within 3 months to 6 months, etc. The reason why we chose a cut-off of 1 year is because we found that the survival rate decrease fast within 1 year in these patients with advanced disease (Supplementary Fig. 1). Logistic regression was used to determine clinical and all related predictors of death within 1-year. In univariate logistic analysis, we further dichotomized the continuous variables according to the optimal cutoff point that was determined at the point of highest accuracy for predicting death within 1 year by receiver operating characteristic (ROC) curve analyses. Kaplan–Meier analyses were done to show the association of hemoglobulin and SII with OS and compared using the log-rank test. When more than one variable was significant in univariate analyses, each significant variable (*p* < 0.05) in the univariate analyses were selected for a multivariate analysis using a forward stepwise method due to multi-colinearities between the parameters. A nomogram was generated to predict the death within 1 year of this patient population. The c-index was used as a measure of discriminative ability of the model. We calculated the nomogram total points for each patient and separated the patients into two groups using the optimal cutoff point of highest accuracy for predicting death within 1 year by ROC curve analysis. The Kaplan–Meier survival curve was done to evaluate separation of survival outcomes of the two groups and compared using the log-rank test. All statistical analyses were done in Stata software, version 12.0 (Stata Corp. LP, College Station, TX).

## Results

One hundred and forty-seven patients were included in this study. Patients’ and disease characteristics were presented in Table 1. The mean age at diagnosis was 61.7 (range 36–93, SD 9.9) years; this cohort included 139 men (95%) and 8 women (5%). The median follow-up period was 599 days. Most patients had stage IV disease (87%, 128/147), and 19 patients (13%) were in the stage III disease. The primary disease located at tonsil in 94 (64%), soft palate in 12 (8%), tongue base in 14 (10%) and pharyngeal wall in 27 (18%) patients. There were 13 (9%) patients had past history of treated cancer and 21(14%) patients had syn- or meta-chronous cancer. Regarding the failure pattern after the completion of treatment, these patients had disease persistence rate of 35% (51/147) and disease recurrence (including local, regional, and distant) rate of 27% (40/147). The 1-year and 2-year overall death rates for all patients was 29% (95% CI: 23–37%) and 52% (95% CI: 44–60%), respectively (Supplementary Fig. 1).

**Table 1:** Characteristics and clinicopathological parameters of the recruited advanced oropharyngeal cancer patients

	N (%) or mean ± SD (range) or median (range, IQR)
Age (years)	61.7 ± 9.9(36–93)
Gender (Male/Female)	139(95%)/8(5%)
T classification (T1 + 2/ T3 + 4)	50(33%)/97(67%)
N classification (N0/N1/N2/N3)	12(8%)/26(18%)/92(63%)/17(11%)
Stage (III/ IVa/ IVb)	19(13%)/96(65%)/32(22%)
Body Height (cm)	166.7 ± 6.1(152–185)
Body Weight (kg)	64.4 ± 12.4(36–100)
BMI (kg/m^2^)	23.2 ± 4.1(12.5–35.4)
Tumor side (Right/Left)	62(42%)/85(58%)
Disease subsite (Tonsil/ Soft palate/ Tongue base/ Pharyngeal wall)	94(64%)/12(8%)/14(10%)/27(18%)
Previous treated cancer	13(9%)
Oral cancer	7
Esophageal cancer	3
Hypopharyngeal cancer	1
Hepatocellular carcinoma	1
Rectal cancer	1
Syn- or Meta-chronous cancer	21(14%)
Esophageal cancer	10
Oral cancer	4
Lung cancer	2
Hepatocellular carcinoma	1
Oral and hypopharyngeal cancer	1
Hypopharyngeal cancer	1
Breast cancer	1
Thyroid cancer	1
Differentiation (Well/Moderately/Poorly/Missing)	3(2%)/37(25%)/37(25%)/70(48%)
HPV (p16) (Positive/Negative/Missing)	29(20%)/39(26%)/79(54%)
Smoking habit (No/Yes)	33(22%)/114(78%)
Hemoglobulin (gm/dl)	13.0 ± 2.2(4.1–16.5)
Lymphocyte count (10^3^/μl)	1.6 ± 0.8(0.5–6.4)
Monocyte count (10^2^/μl)	0.6 ± 0.4(0.2–4.1)
Platelet count (10^3^/μl)	254.2 ± 85.4(91.0–475.0)
Neutrophil count (10^3^/μl)	6.0 ± 2.8(1.2–17.2)
LMR	3.2 ± 1.8(0.5–8.9)
PLR	184.3 ± 102.0(36.7–697.2)
NLR	4.5 ± 2.9(0.9–16.2)
SIRI	264.3 ± 225.3(21.8–1391.4)
SII	1158.2 ± 935.5(133.0–5209.9)
Median follow-up (range, IQR) (days)	599(315–1446, 1131)
Death within 1 year	43(29%)
Disease Persistence	51(35%)
Recurrence	40(27%)
Local	16
Regional	7
Locoregional	1
Distant	20

*LMR* Lymphocyte to monocyte ratio, *PLR* Platelet to lymphocyte ratio, *NLR* Neutrophil to lymphocyte ratio, *SII* Systemic immune inflammation, *SIRI* Systemic inflammation response index, *IQR* Interquartile range

**Fig. 1:**
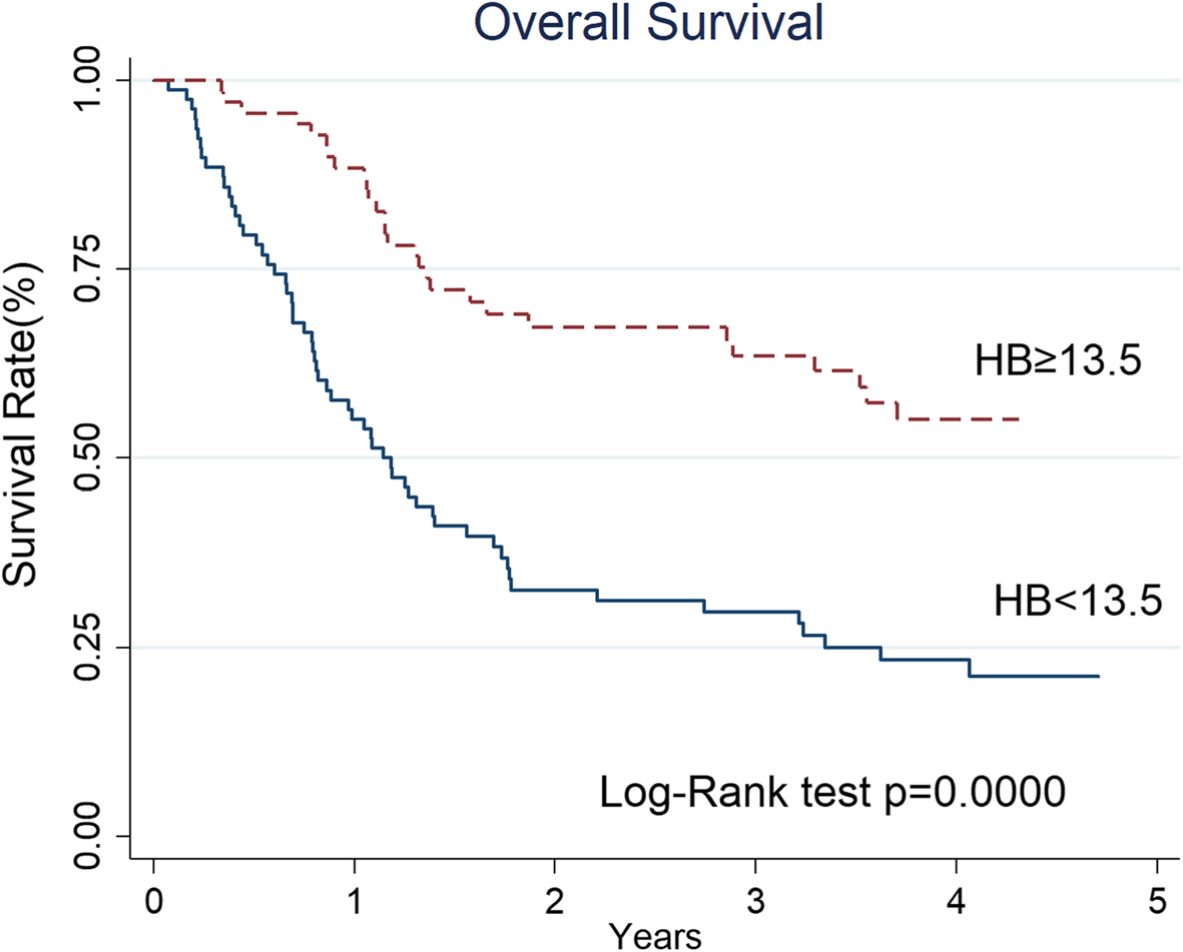
The 1-year and 2-year OS rates for advanced oropharyngeal cancer patients with Hb ≥ 13.5 g/dl versus Hb < 13.5 g/dl were 88 and 67% versus 55 and 33%, respectively

**Fig. 2:**
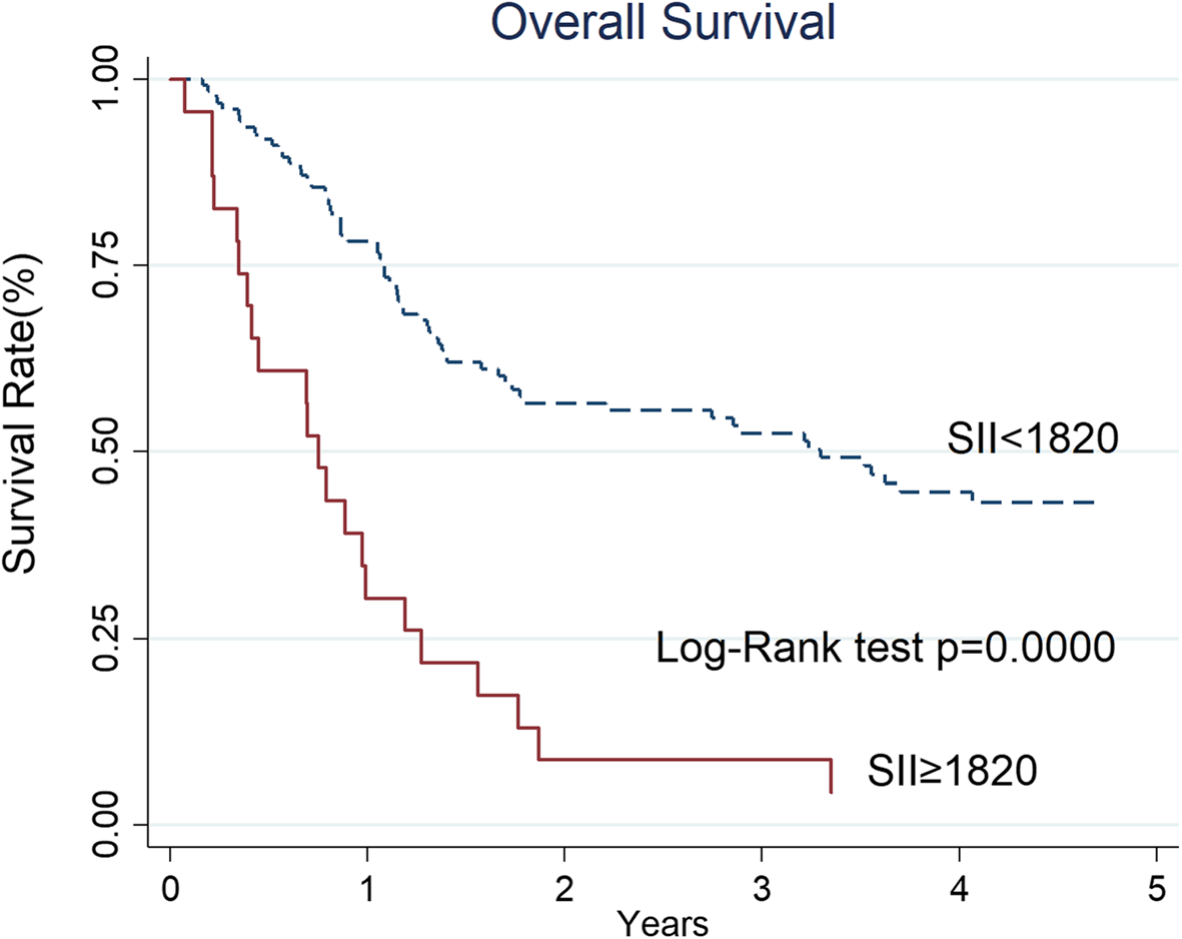
The 1-year and 2-year OS rates for advanced oropharyngeal cancer patients with low SII (< 1820) versus high SII (≥ 1820) were 78 and 57% versus 30 and 9%, respectively

In the univariate analysis, T classification (*p* = 0.004), body height (*p* = 0.015), body weight (*p* = 0.014), body mass index (BMI, *p* = 0.006), Hb (*p* < 0.001), platelet count (*p* < 0.001), neutrophil count (*p* = 0.004), PLR (*p* = 0.047), NLR (*p* = 0.041), SIRI (*p* = 0.004), and SII (*p* < 0.001) were significant risk factors for death within 1-year (Table 2). The multivariate logistic regression analyses using a forward stepwise model adjusted for age and gender (Table 3) showed that Hb < 13.5 g/dl (OR 5.85, 95% CI 2.17–15.75, *p* < 0.001) and SII ≥ 1820 (OR 4.78, 95% CI 1.44–15.85, *p* = 0.011) were independent risk factors for death within 1 year in advanced oropharyngeal cancer patients. The 1-year and 2-year OS of the patients with Hb ≥ 13.5 g/dl versus Hb < 13.5 g/dl was 88 and 67% versus 55 and 33%, respectively (*p* < 0.001, Fig. 1). For patients with low SII (< 1820) versus high SII (≥ 1820), the 1-year and 2-year OS was 78 and 56% versus 30 and 9%, respectively (*p* < 0.001, Fig. 2).

**Table 2:** Univariate analyses of clinicopathological factors between patients with death within 1-year and the others in advanced oropharyngeal cancer patients

Variables		Early death patients (*n* = 43)	Other patients (*n* = 104)	*P* value
Age (years)	≥ 60	20(47%)	62(40%)	0.146
	< 60	23(53%)	42(60%)	
Gender	Male	42(98%)	97(93%)	0.284
	Female	1(2%)	7(7%)	
T	T1 + 2	7(16%)	43(41%)	0.004*
	T3 + 4	36(84%)	61(59%)	
N	N0 + 1 + 2a	14(33%)	51(49%)	0.067
	N2b + 2c + 3	29(67%)	53(51%)	
Stage	III	4(9%)	15(14%)	0.400
	IV	39(91%)	89(86%)	
Disease subsite	Tonsil	25(58%)	69(66%)	0.097
	Soft palate	2(5%)	10(10%)	
	Tongue base	3(7%)	11(11%)	
	Pharyngeal wall	13(30%)	14(13%)	
Previous treated cancer	No	40(93%)	94(90%)	0.608
	Yes	3(7%)	10(10%)	
Syn- or Meta-chronous cancer	No	36(84%)	90(87%)	0.657
	Yes	7(16%)	14(13%)	
Differentiation	Well	0(0%)	3(5%)	0.370
	Moderately	8(18%)	29(27%)	
	Poorly	11(26%)	26(25%)	
	Missing	24(56%)	46(43%)	
HPV (P16)	Positive	7(16%)	32(31%)	0.086
	Negative	7(16%)	22(21%)	
	Missing	29(68%)	50(48%)	
Smoking habit	No	10(23%)	23(22%)	0.943
	Yes, quitted	12(28%)	27(26%)	
	Yes, persistent	21(49%)	54(52%)	
Body Height (cm)	≥163	38(88%)	72(69%)	0.015*
	< 163	5(12%)	32(31%)	
Body Weight (kg)	≥60	22(51%)	73(70%)	0.014*
	< 60	21(49%)	31(30%)	
BMI (kg/m^2^)	≥22	18(42%)	69(66%)	0.006*
	< 22	25(58%)	35(34%)	
Hemoglobulin (gm/dl)	≥ 13.5	8(19%)	61(59%)	0.000*
	< 13.5	35(81%)	43(41%)	
Lymphocyte count (10^3^/μl)	≥1.85	16(37%)	27(26%)	0.173
	< 1.85	27(63%)	77(74%)	
Monocyte count (10^2^/μl)	≥0.56	21(49%)	43(41%)	0.405
	< 0.56	22(51%)	61(59%)	
Platelet count (10^3^/μl)	≥239	34(79%)	44(42%)	0.000*
	< 239	9(21%)	60(58%)	
Neutrophil count (10^3^/μl)	≥7.5	17(40%)	28(17%)	0.004*
	< 7.5	26(60%)	86(83%)	
LMR	≥1.9	30(70%)	80(77%)	0.363
	< 1.9	13(30%)	24(23%)	
PLR	≥157	28(65%)	49(47%)	0.047*
	< 157	15(35%)	55(53%)	
NLR	≥5.1	19(44%)	28(27%)	0.041*
	< 5.1	24(56%)	76(63%)	
SIRI	≥553.7	10(23%)	7(7%)	0.004*
	< 553.7	33(77%)	97(93%)	
SII	≥1820	16(37%)	7(7%)	0.000*
	< 1820	27(63%)	97(93%)	

*: *p* < 0.05

*LMR* lymphocyte to monocyte ratio; *PLR* platelet to lymphocyte ratio; *NLR* neutrophil to lymphocyte ratio; *SII* systemic immune inflammation; *SIRI* systemic inflammation response index

**Table 3:** Univariate and stepwise multivariate logistic regression analyses of clinicopathological factors that related to death within 1-year in advanced oropharyngeal cancer patients

	Univariate			Multivariate				
	OR	95% CI		*p*-value	OR	95% CI		*p*-value
Gender								
Female	Ref.				Ref.			
Male	3.031	0.362	25.412	0.307	2.386	0.249	22.817	0.450
Age								
< 60y	Ref.				Ref.			
≥ 60y	1.698	0.830	3.473	0.147	2.200	0.881	5.495	0.091
T								
T1 + 2	Ref.							
T3 + 4	3.625	1.476	8.906	0.005*				
N								
N0 + 1	Ref.				Ref.			
N2	1.202	0.500	2.892	0.681	1.050	0.292	3.774	0.940
N3	3.625	1.080	12.167	0.037*	1.552	0.267	9.023	0.624
Stage								
III	Ref.				Ref.			
IVa	0.925	0.275	3.109	0.900	0.575	0.104	3.172	0.525
IVb	6.250	1.678	23.275	0.006*	2.700	0.391	18.629	0.314
Subsite								
Tonsil	Ref.							
Soft palate	0.552	0.113	2.695	0.463				
Tongue base	0.753	0.194	2.921	0.681				
Pharyngeal wall	2.563	1.060	6.196	0.037*				
Side								
R	Ref.							
L	1.019	0.496	2.092	0.960				
Previous treated cancer								
None	Ref.							
Yes	0.705	0.184	2.698	0.610				
Syn−/Meta-chornous cancer								
None	Ref.							
Yes	1.250	0.466	3.351	0.657				
Differentiation								
Well	Ref.							
Moderately + Poorly	0.705	0.345	1.441	0.338				
HPV (p16)								
Positive	Ref.							
Negative	1.455	0.447	4.733	0.534				
Smoking habit								
No	Ref.							
Yes, quitted	1.022	0.374	2.798	0.966				
Yes, persistence	0.894	0.365	2.194	0.808				
BMI								
< 22	Ref.							
≥ 22	0.365	0.176	0.758	0.007*				
Hemoglobulin (gm/dl)								
≥ 13.5	Ref.				Ref.			
< 13.5	6.206	2.622	14.689	0.000*	5.846	2.169	15.754	0.000*
Lymphocyte count (10^3^/μl)								
≥ 1.85	Ref.							
< 1.85	1.690	0.792	3.606	0.175				
Monocyte count (10^2^/μl)								
< 0.56	Ref.							
≥ 0.56	1.354	0.663	2.765	0.405				
Platelet count (10^3^/μl)								
< 239	Ref.							
≥ 239	5.152	2.243	11.830	0.000*				
Neutrophil count (10^3^/μl)								
< 7.5	Ref.							
≥ 7.5	3.124	1.411	6.917	0.005*				
LMR								
< 1.9	Ref.							
≥ 1.9	0.692	0.313	1.533	0.364				
PLR								
< 157	Ref.							
≥ 157	2.100	1.004	4.373	0.049*				
NLR								
< 5.1	Ref.							
≥ 5.1	2.149	1.024	4.511	0.043*				
SIRI								
< 553.7	Ref.							
≥ 553.7	4.199	1.479	11.922	0.007*				
SII								
< 1820	Ref.				Ref.			
≥ 1820	8.212	3.066	21.995	0.000*	4.777	1.440	15.846	0.011*

*: *p* < 0.05

*LMR* Lymphocyte to monocyte ratio, *PLR* Platelet to lymphocyte ratio, *NLR* Neutrophil to lymphocyte ratio, *SII* Systemic immune inflammation, *SIRI* Systemic inflammation response index

**Fig. 3:**
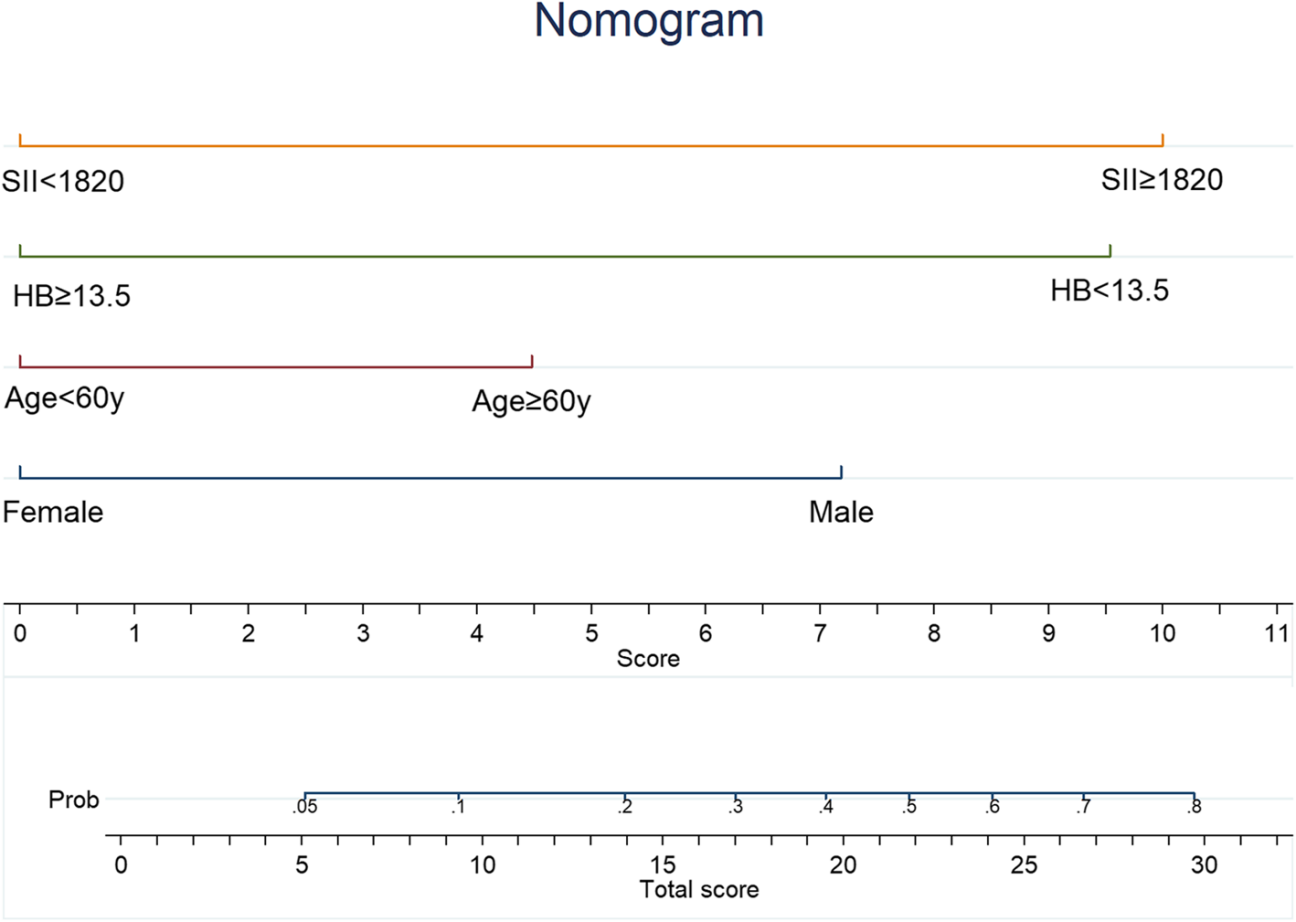
Nomogram of death within 1-year in advanced oropharyngeal cancer patients

**Fig. 4:**
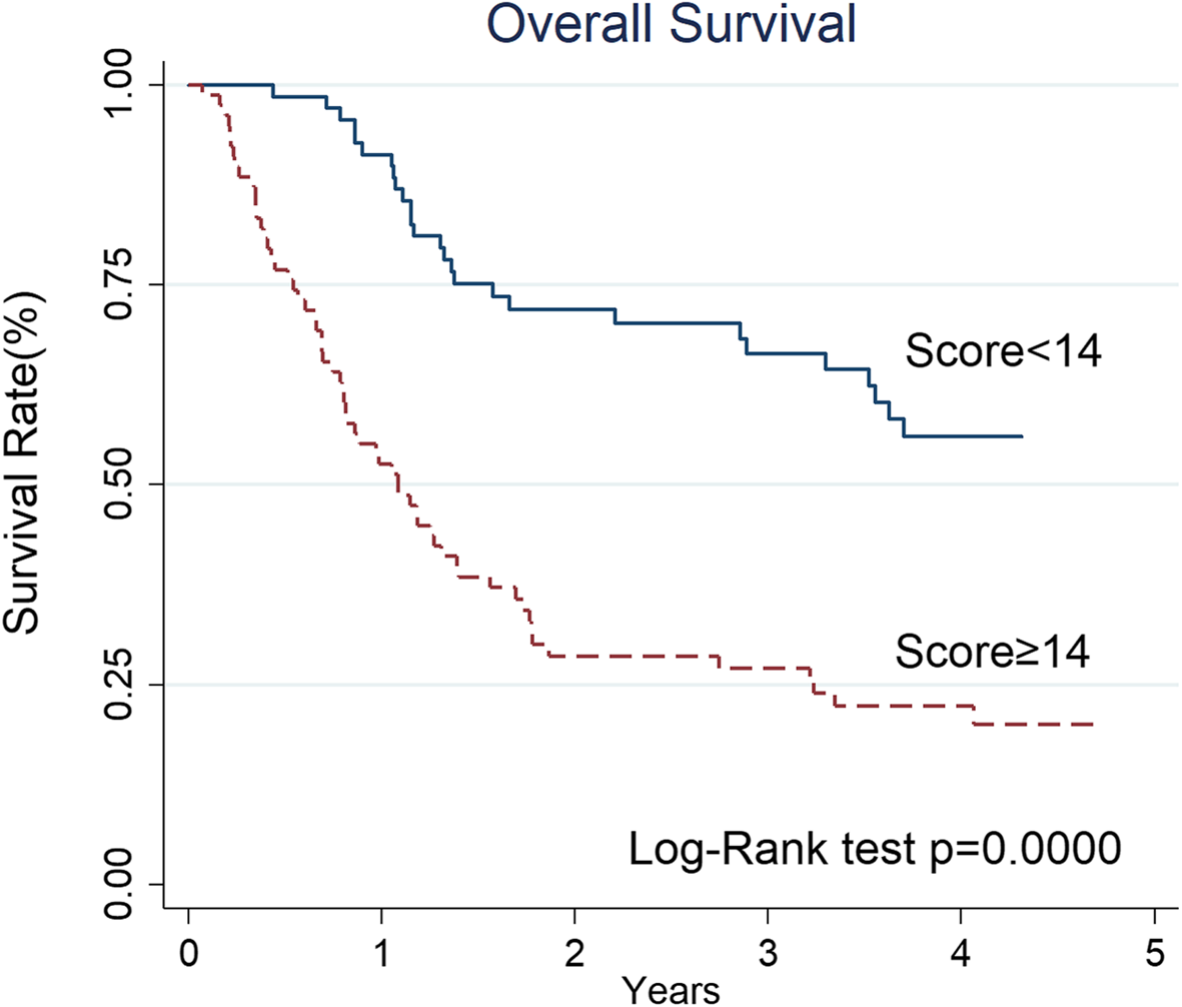
The 1-year and 2-year OS rates for advanced oropharyngeal patients with low nomogram score (< 14) versus high nomogram score (≥ 14) were 91 and 71% versus 53 and 29%, respectively

The independent risk factors plus age and gender were used to create a nomogram (Fig. 3) based prognostic score for early death probability. The nomogram lets us find the early death prediction rapidly and straightforwardly according to a patient’s individual features. The usage of the nomogram was illustrated to as follows: 1. Draw a vertical line for the observed value of each predictive variable to the “Score” line; 2. Sum the values on the “Score” line to obtain the total scores; 3. Draw a vertical line from the “Total score” up to the probability line to predict the rate of death within 1 year. The c-index of the model was 0.75 (95%CI 0.66–0.83). Figure 4 illustrated good discrimination ability for two groups based on partitioning patients’ total scores. For patients with low nomogram score (< 14) versus high nomogram score (≥ 14), the 1-year and 2-year OS rates were 91 and 71% versus 53 and 29%, respectively. (*p* < 0.001).

## Discussion

We found both the pre-treatment Hb concentration and SII were independent prognostic parameters associated with death within 1-year in advanced oropharyngeal cancer patients treated with concurrent chemoradiation in our series (Table 3). These two markers are easily available and low-priced markers of systemic inflammation that can lead us clinical decisions regarding treatment outcome and survival. We observed that patients with Hb ≥ 13.5 g/dl had a significantly higher chance of survival than those with Hb < 13.5 g/dl; the risk of death within 1-year in high Hb group was significantly lower than that in low Hb group (*OR* 5.85, 95% *CI* 2.17–15.75). The adverse impact in patients with low Hb before treatment has been well documented in head and neck cancer patients treated with (chemo)radiation [5, 6, 8, 10, 15]. The mechanism underlying poor prognosis remains discussed. It is suggested that low Hb concentration may exacerbate the preexisting tumor hypoxia and, hence, impeding the response of tumor cells to cytotoxic therapy [23]. Furthermore, the so-called oxygen enhanced ratio is that the ability of cancer cell eradication induced by ionizing radiation can be heightened by oxygen up to 2.5–3 times [24, 25]. However, blood transfusions and erythropoiesis-stimulating agents are once considered promising but have been proven to be of lower or no survival benefits in some clinical researches [9, 24, 26, 27]. As a result, hypoxia may be related to treatment results because of diminishing radiation efficacy, as well as being an indicator of disease aggressiveness.

Inflammatory state and immune status have been recognized as hallmarks of head and neck oncogenesis, and many clinical trials have now shown peripheral blood biomarkers to be associated with treatment outcomes [11–15, 17, 21, 22, 28, 29]. In the current study, we compared the absolute neutrophil, platelet, monocyte, and platelet counts, as well as the combinations (LMR, PLR, NLR, SIRI and SII) at the same time to determine which one to be the most predictive of death within 1 year in this advanced oropharyngeal cancer population. The chance of death within 1 year is significantly higher in patients with SII ≥ 1820 compared with those < 1820 in advanced oropharyngeal cancer (*OR* 4.78, 95% *CI* 1.44–15.85). The survival rate in high SII group dropped to 30% in 1-year and was reduced even further to 9% in 2-year.

The true mechanism between a high pre-treatment SII and a poor treatment outcome remains uncertain. Tumor-associated neutrophils promote tumor growth and metastasis by triggering regional immune responses that increase circulating chemokines and cytokines, promote tumor angiogenesis, and lead to proliferation and infiltration [17, 19, 20]. Platelet are recognized as a stimulator of proangiogenic factors, platelet derived growth factor, and fibroblast growth factor, which promote tumor invasion and growth [21, 30]. On the other hand, lymphocytes, plays a role in blocking tumor growth by cytotoxic activity [14, 18]. As revealed in this study, the SII reflects the host inflammation and immune status better than singular parameters or the other immune ratios (LMR, PLR, NLR, and SIRI).

The traditional chemoradiotherapy does not provide an effective disease control for patients with low Hb and/or high SII. In this study, the predictive model of the nomogram provides graphical depiction that can be used to estimate the probability of death within 1 year for an individual patient. To the best of our knowledge, the current study is the first study to use the nomogram to predict the death within 1-year in advanced oropharyngeal cancer patients treated with chemoradiation. Table 4 showed the failure pattern in patients with high and low nomogram score. The disease persistence/recurrence rate for patients with nomogram scores that were ≥ 14 and < 14 was 73 and 28%, respectively (*p* < 0.001). According to our results, nomogram score ≥ 14 increased the risk of disease persistence/recurrence by more than 2.5-fold. An ideal tool to be predictive of the responders and non-responders shall be easily available, reproducible and cheap. The nomogram presented in this study, if validated in an external independent trial, can become a good predictive model and can has potential for identifying patients suited in need of new treatment choices or innovative therapeutic combinations that strengthen the current treatment.

**Table 4:** Disease persistence/recurrence according to the nomogram score in patients with advanced oropharyngeal cancer

		Disease persistence/ recurrence (*n* = 83)	None (*n* = 64)	*p*-value
Nomogram score	< 14	26(28%)	43(62%)	0.000
	≥14	57(73%)	21(27%)	

Several limitations of our study should be addressed. First, this is a single-institution retrospective study, unnoticed or unavoidable selection bias might have played a role and the practice patterns might vary among different institutions. For example, a possible bias of the study may be the fact that part of the sample of test subjects received adjuvant chemotherapy. Second, HPV status was not related to death within 1 year in advanced oropharyngeal cancer patients that demonstrated in Table 3. The possible reason is that not all tumors had HPV testing in this series and this factor could influence the outcome. In our country, the prevalence of HPV-positive oropharyngeal cancer was 28.4% [31]. The smoking prevalence in the current study was 78%. As a result, most of our advanced oropharyngeal cancer patients were supposed to be driven by traditional carcinogen. There is another possibility that the occurrence of death within 1-year in these patients with large tumor burden is mainly related to the inflammatory status in despite of their HPV status. Independent external validation trial utilizing large samples and high level published data is warranted to validate the predictive nomogram in advanced oropharyngeal cancer patients. The strength of this study is its large cohort of homogeneous patients and treatment (advanced oropharyngeal cancer treated with chemoradiotherapy) in a single center. Single center cohorts have the benefit of the capability to do in depth chart review and validate recorded data. We can obtain comprehensive information about pretreatment laboratory values in all patients.

In conclusion, the Hb and SII are important prognostic features for advanced oropharyngeal cancer and should be evaluated in routine pretreatment assessments. Nomogram that combined these two factors may aid patient selection or stratification.

for clinical trial and indicates the need of establishment of more effective treatment regimens than the traditional therapy in high risk patient group.

## Supplementary Information

**Additional file 1: Supplementary Fig. 1**. The 1-year and 2-year overall mortality rates for advanced oropharyngeal cancer patients were 29% (95% CI: 23–37%) and 52% (95% CI: 44–60%), respectively.

## Data Availability

The datasets used and analysed during the current study are available from the corresponding author on reasonable request.
