# Supplementary material for: A predictive model for advanced oropharyngeal cancer patients treated with chemoradiation
Source: BMC Cancer. 2022 Jun 5;22:615. doi: 10.1186/s12885-022-09732-9 (PMC9167527; doi:10.1186/s12885-022-09732-9)

Supplementary Fig. 1. The 1-year and 2-year overall mortality rates for advanced oropharyngeal cancer patients were 29% (95% CI: 23-37%) and 52% (95% CI: 44-60%), respectively.


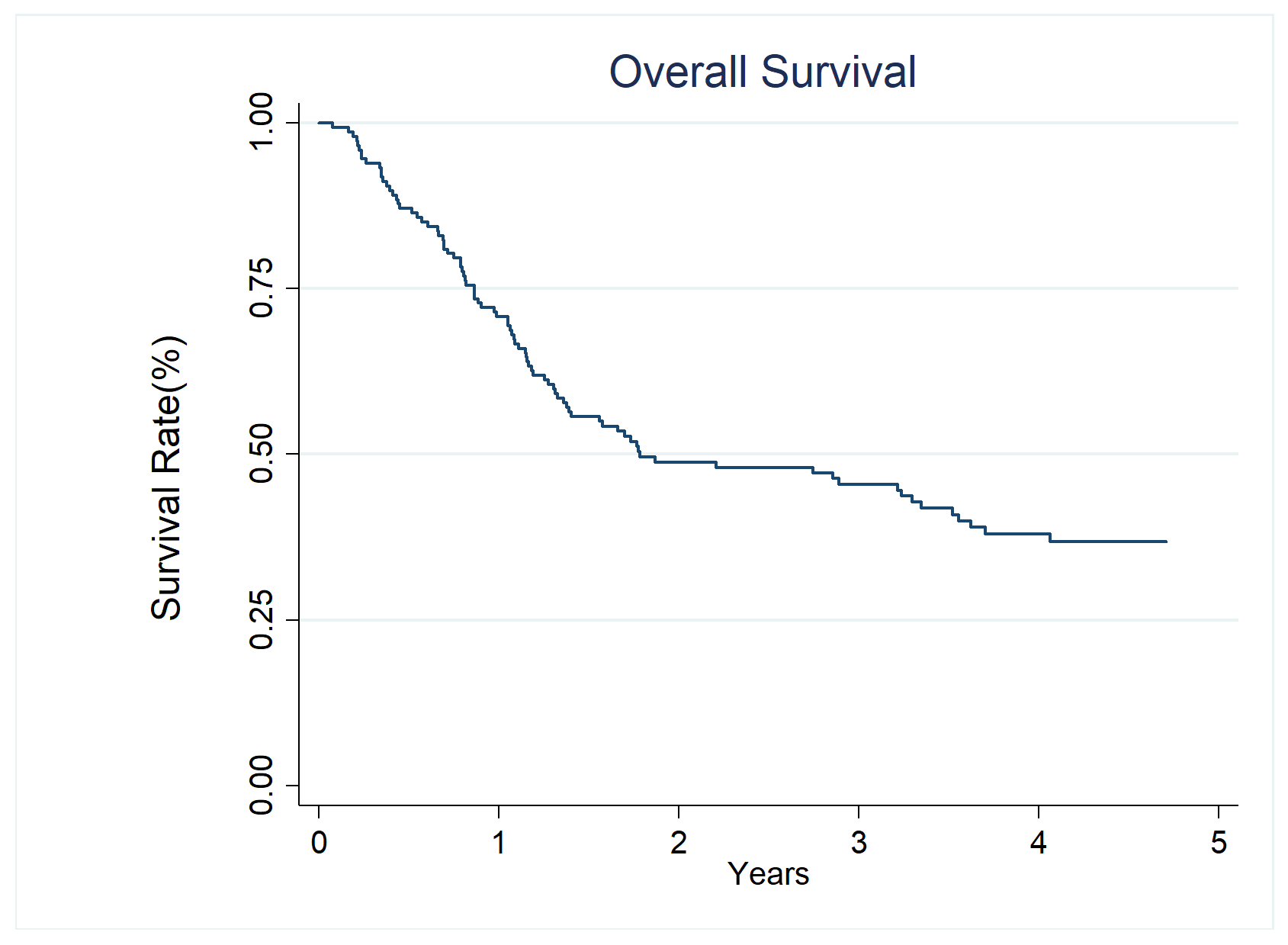

Supplement: Supplementary file 1 — Additional file 1: Supplementary Fig. 1. The 1-year and 2-year overall mortality rates for advanced oropharyngeal cancer patients were 29% (95% CI: 23–37%) and 52% (95% CI: 44–60%), respectively. [file 12885_2022_9732_MOESM1_ESM.docx]
